# Association between depressive-symptom trajectories and cognitive function in the late middle-aged and older population: results of the Korean Longitudinal Study of Ageing

**DOI:** 10.1038/s41598-019-44158-7

**Published:** 2019-05-24

**Authors:** Dong-Woo Choi, Kyu-Tae Han, Jooeun Jeon, Sung-In Jang, Seung Ju Kim, Eun-Cheol Park

**Affiliations:** 1 Department of Public Health, Graduate School, Yonsei University, Seoul, Republic of Korea; 2 Institute of Health Services Research, Yonsei University, Seoul, Republic of Korea; 3 Hospice & Palliative Care Branch, National Cancer Control Institute, National Cancer Center, Goyang, South Korea; 4 Department of Preventive Medicine, Yonsei University College of Medicine, Seoul, Republic of Korea; 5 Department of Nursing, College of Nursing, Eulji University, Seongnam, Gyeonggi-do Republic of Korea

**Keywords:** Geriatrics, Epidemiology, Epidemiology

## Abstract

This study investigated the association between depressive symptoms and cognitive function according to four different trajectories of depressive symptoms in the late middle-aged and older South Korean population. Panel data from the Korean Longitudinal Study of Ageing were analyzed. We used latent class trajectory models to identify four trajectories of depressive symptoms. We performed linear mixed-effects regression analysis to assess associations between depressive-symptom trajectories and MMSE scores. Of 4,374 participants, 18.4%, 4.9%, 55.2%, and 21.5% were classified as having a low, increasing, moderate declining, and high depressive-symptom trajectory, respectively. Individuals with an increasing trajectory (β = −0.729, *P* ≤ 0.001), moderate trajectory (β = −0.278, *P* = 0.003), and high trajectory (β = −1.605, *P* ≤ 0.001) had lower MMSE scores compared with those in the low trajectory group. These relationships were particularly strong among women; individuals who were physically inactive; those who were separated, divorced, or single; and those with hypertension or cerebrovascular disease. Each trajectory group for depressive symptoms was associated with cognitive decline. Moreover, female, physically inactive, and single individuals, as well as those with hypertension and cerebrovascular disease should be particularly mindful of their mental and physical health to prevent cognitive decline.

## Introduction

In South Korea, dementia has become a health priority because of the rapidly aging population. Indeed, South Korea already became aged society in 2017 and it is predicted that 38.2% of the Korean population is likely to be aged 65 years or older by 2050, which is much higher than that forecasted for other developed countries^1^. Cognitive function decline is the most common health problem among the elderly. Moreover, early-onset dementia is not uncommon among 45- to 65-year-old individuals and reportedly accounts for approximately one-third of all patients with dementia in developed countries^2–4^. Age-related cognitive decline is highly variable with little or no decline in many individuals but slight to precipitous decline in others^5–7^. It is associated with several factors, such as mental disease, chronic disease, and environmental factors^8,9^. Among these, depression is a well-known risk factor and a major worldwide public health concern^10^.

Depressive symptoms are affected by an individual’s subjective mood at the time of assessment and can vary throughout one’s life^11,12^. Moreover, patterns of symptoms vary among individuals, with some experiencing chronic depression, others experiencing sudden increases in depressive symptoms in reaction to life events, and others experiencing periods of relative remission from depression that may be punctuated by intermittent relapses of symptoms. These different patterns of change in depressive symptoms might be affected by several risk factors, including an individual’s environment and health behaviors, as well as biological, genetic, and social factors^13–15^.

Although some previous studies have noted the importance of considering different patterns of depressive symptoms, most studies investigating the relationship between depression and cognitive function have assessed depressive symptoms cross-sectionally or as a repeated measure without considering the individual characteristics of changes in depressive symptoms^16^. Moreover, in the general population, individuals experience several changes in the course of their depressive symptoms, each of which might be related to different risk factors or outcomes.

Approaches that consider the different patterns in depressive symptoms could aid health policy makers and health professionals to develop optimized health policies for dementia by considering individual characteristics for more efficient use of limited health resources. Thus, this study investigated the association between depressive symptoms and cognitive function according to four different trajectories of depressive symptoms in the late middle-aged and older South Korean population.

## Methods

### Study population

We analyzed panel data from the Korean Longitudinal Study of Ageing (KLoSA) waves 2 (2008) to 6 (2016). The KLoSA data included those of participants aged 45 years or older living in households selected by multistage stratified probability sampling to be representative of the nation. Follow-up examinations conducted via home interviews took place every 2 years.

In the current study, we included all participants enrolled from 2008 to 2016. However, we excluded individuals with suspected dementia (represented by a Mini-Mental State Examination (MMSE) score lower than 17 in 2008^17^). We also excluded those who did not provide responses for relevant covariates when completing the questionnaires of the KLoSA. Accordingly, 4,374 participants were included in this study.

### Variables

The primary outcome was the MMSE score. The MMSE is a global measurement of cognition, with domains for orientation, concentration, language, praxis, and immediate and delayed memory. The Korean version of this test was administered according to standardized procedures, with a total score ranging from 0 to 30 where lower scores represent poor cognitive functioning^18^.

We focused primarily on depressive symptoms by using the 10-item version of the Center for Epidemiology Depression Scale (CES-D10). The CES-D10 has excellent properties for use as a screening tool to identify major depressive symptoms in older adults^19^.

We assessed several covariates, including sex, age, educational level (“elementary school or under,” “middle school,” “high school,” or “university or above”), household assets quartile (1st, 2nd, 3rd, or 4th quartile), employment status (“employed” or “unemployed”), marital status (“married” or “separated, divorced, or single”), body mass index (BMI, “pre-obesity or obesity,” “overweight,” or “normal or underweight”), physical activity (“at least once a week” or “none”), alcohol consumption (“drinker,” “ex-drinker,” or “non-drinker”), smoking behavior (“current smoker,” “ex-smoker,” or “non-smoker”), and the presence of hypertension or cerebrovascular disease (previously diagnosed by a doctor).

### Statistical analysis

We used latent class trajectory models to identify trajectories of depressive symptoms according to the CES-D10 over the follow-up period. This is a specialized form of finite mixture modelling, which describes the course of depressive symptomatology through a regression function using continuous latent growth factors (intercept, slope, quadratic slope, and cubic slope). The intercept represents the level of depressive symptoms at baseline. The change in depressive symptoms over time is accounted for by one or more growth factors such as a linear, quadratic, or cubic slope.

We calculated the posterior probabilities for each trajectory taking into account the participant’s age (at baseline of depressive symptoms), educational level, BMI, smoking status, alcohol status, employment status, educational level, and household assets. Subsequently, we assigned participants post hoc to the trajectory with the highest probability. We estimated the best-fitting trajectory based on a minimum Bayesian information criterion. To facilitate interpretability, we labeled trajectories based on their modeled plot patterns as “low CES-D trajectory,” “increasing CES-D trajectory,” “moderate declining CES-D trajectory,” and “high CES-D trajectory.”

We conducted analysis of variance and chi-square tests for the descriptive statistics of the trajectories. Thereafter, we performed linear mixed-effects regression to assess the relationship between the depressive-symptom trajectory groups and the MMSE score after adjusting for covariates, including sex, age, education level, household assets, employment status, marital status, BMI, physical activity, alcohol consumption, smoking, hypertension, and cerebrovascular disease. The linear mixed-effects regression examined within-individual changes and between-individual differences in change over time. Thereafter, we conducted subgroup analyses for sex, marital status, physical activity, hypertension, and cerebrovascular disease using the linear mixed-effects model.

Finally, we stratified the follow-up period into 2008 to 2010, 2010 to 2012, 2012 to 2014, and 2012 to 2016 to explore the possibility of reverse causality. All statistical analyses were conducted with SAS version 9.4 (SAS institute, Inc., Cary, NC, USA). Statistical significance was defined as *P* < 0.05.

### Ethics approval and consent to participate

The Korean Longitudinal Study of Ageing data are openly published. Thus, ethical approval was not required for this study. This study did not require informed consent from the participants, as their information was fully anonymized and unidentified prior to analysis.

## Results

**Figure 1:**
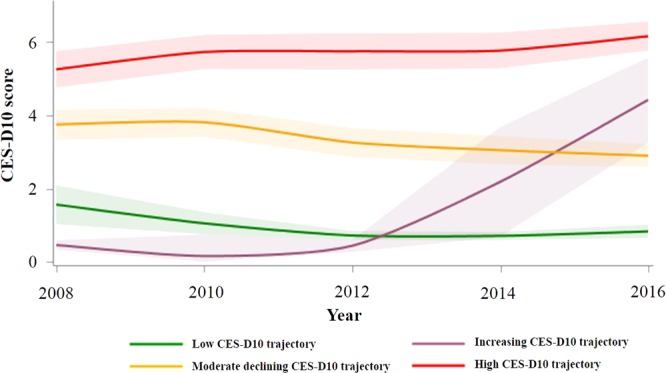
Trajectories for the CES-D10 scores between 2008 and 2016 in each group.

Figure 1 presents the four trajectories of depressive symptoms between 2008 and 2016 in the 4,374 participants. Participants were classified as maintaining a low CES-D10 score throughout the follow-up period (n = 804, 18.4%), beginning with a low CES-D10 score that rapidly increased (n = 215, 4.9%), having a moderate declining CES-D10 score (n = 2,413, 55.2%), or maintaining a high CES-D10 score (n = 942, 21.5%).

Table 1 shows the general characteristics of the baseline population according to their identified trajectories. In each of the four trajectories, 49%, 47%, 43%, and 45% of the respective participants were male. The greatest proportion of married individuals were in the low CES-D trajectory group (n = 714, 88.8%), followed by the increasing CES-D trajectory group (n = 186, 86.5%), moderate declining CES-D trajectory group (n = 2,025, 83.9%), and high CES-D trajectory group (n = 712, 75.6%). The mean CES-D10 scores for each trajectory were 0.4 ± 0.9 (low trajectory), 0.8 ± 1.7 (increasing CES-D trajectory), 3.1 ± 2.3 (moderate declining CES-D trajectory), and 6.3 ± 1.9 (high CES-D trajectory). The overall mean CES-D10 score was 2.9 ± 2.7. The mean MMSE scores were 27.0 ± 2.8, 25.6 ± 4.0, 26.3 ± 3.2, and 24.5 ± 4.2 for the low, increasing, moderate declining, and high trajectories, respectively, and the overall mean MMSE score was 26.1 ± 4.1.

**Table 1:** Baseline characteristics of study population.

Variables	Total (N = 4,374)	Low CES-D10 trajectory (N = 804)	Moderated declining CES-D10 trajectory (N = 2,413)	Increasing CES-D10 trajectory (N = 215)	High CES-D10 trajectory (N = 942)	*P*-value
**CES-D10 score** ^**†**^	3.0 ± 2.7	1.0 ± 1.6	3.3 ± 2.5	0.5 ± 1.0	5.5 ± 2.4	
**MMSE score** ^**†**^	26.0 ± 3.8	27.7 ± 2.6	27.2 ± 2.7	27.0 ± 3.0	25.6 ± 3.2	
**Sex**						<0.001
Men	1,968	48.8	43.3	47.0	45.5	
Women	2,406	51.2	56.7	53.0	54.5	
**Age (years)**						<0.001
45–54	1,362	35.1	31.2	33.5	27.0	
55–64	1,540	37.7	35.0	37.2	33.2	
65–74	1,180	22.3	27.7	24.7	29.7	
≥75	292	5.0	6.1	4.7	10.1	
**Educational level**						<0.001
Elementary school or under	1,703	35.3	39.3	38.1	41.2	
Middle school	819	19.0	18.7	16.3	19.0	
High school	1,444	34.6	33.4	37.2	29.7	
University or above	408	11.1	8.5	8.4	10.1	
**Household assets quartile**						0.690
1st quartile	949	20.4	19.9	20.9	27.7	
2nd quartile	1,051	25.1	24.8	26.5	20.6	
3rd quartile	1,193	26.7	28.8	25.6	24.3	
4th quartile	1,181	27.7	26.6	27.0	27.4	
**Employment status**						<0.001
Employed	2,179	52.6	50.1	52.1	46.2	
Unemployed	2,195	47.4	49.9	47.9	53.8	
**Marital status**						0.064
Married	3,760	89.9	87.2	89.3	78.8	
Separated, divorced, or single	614	10.1	12.8	10.7	21.2	
**BMI**						0.335
Pre-obesity or obesity	1,000	21.0	23.0	28.4	22.9	
Overweight	1,427	32.0	33.4	33.5	31.0	
Normal or underweight	1,947	47.0	43.6	38.1	46.1	
**Physical activity**						0.001
Yes	1,786	44.3	42.4	37.2	34.7	
No	2,588	55.7	57.6	62.8	65.3	
**Alcohol behavior**						0.131
Drinker	1,802	42.3	41.3	36.7	41.1	
Ex-drinker	340	7.6	8.1	5.1	7.7	
Non-drinker	2,232	50.1	50.6	58.1	51.2	
**Smoking behavior**						0.259
Current smoker	812	19.9	17.6	16.7	20.3	
Ex-smoker	532	11.9	12.6	11.2	11.4	
Non-smoker	3,030	68.2	69.7	72.1	68.4	
**Hypertension**						0.558
Present	1,242	25.9	28.6	24.2	31.0	
Absent	3,132	74.1	71.4	75.8	69.0	
**Cerebrovascular disease**						<0.001
Present	93	0.5	2.3	0.9	3.4	
Absent	4,281	99.5	97.7	99.1	96.6	
**Total**	4,374	18.4	55.2	4.9	21.5	

^†^Mean and standard deviation (SD) of the continuous independent variables in this study.

^‡^Categorical variables are presented as frequencies and percentages.

Table 2 presents the results of the linear mixed-effects regression to investigate the association between trajectory groups of depressive symptoms and MMSE scores. We observed that there was a statistically significant inverse relationship between the CES-D10 scores and MMSE scores (*β* = −0.124, *P* ≤ 0.001). Among the CES-D trajectory groups, the increasing CES-D trajectory (*β* = −0.729, *P* ≤ 0.001), moderate declining CES-D trajectory (*β* = −0.278, *P* = 0.003), and high CES-D trajectory (*β* = −1.605, *P* ≤ 0.001) groups were associated with a lower MMSE score than was the low CES-D trajectory group. We did not observe any significant differences between the sexes. However, separated, divorced, or single individuals had lower MMSE scores than did married participants (*β* = −0.278, *P* = 0.001). Meanwhile, participants who engaged in physical activity had higher MMSE scores than did those who did not (*β* = 0.513, *P* ≤ 0.001). Finally, participants with hypertension (*β* = −0.267, *P* ≤ 0.001) or cerebrovascular disease (*β* = −1.339, *P* ≤ 0.001) had lower MMSE scores than did those without these conditions.

**Table 2:** Results of the association between the trajectory groups of depressive symptoms and MMSE scores in a mixed-effects regression analysis.

Variables	MMSE score		
	β	SE	*P*-value
**Trajectory groups**			
Low CES-D10 trajectory	Ref.		
Moderate declining CES-D10 trajectory	−0.278	0.094	0.003
Increasing CES-D10 trajectory	−0.729	0.173	<0.001
High CES-D10 trajectory	−1.605	0.119	<0.001

^†^Analysis was adjusted for the following covariates: sex, age, education level, household assets, employment status, marital status, BMI, physical activity, alcohol consumption, smoking, hypertension, and cerebrovascular disease.

**Figure 2:**
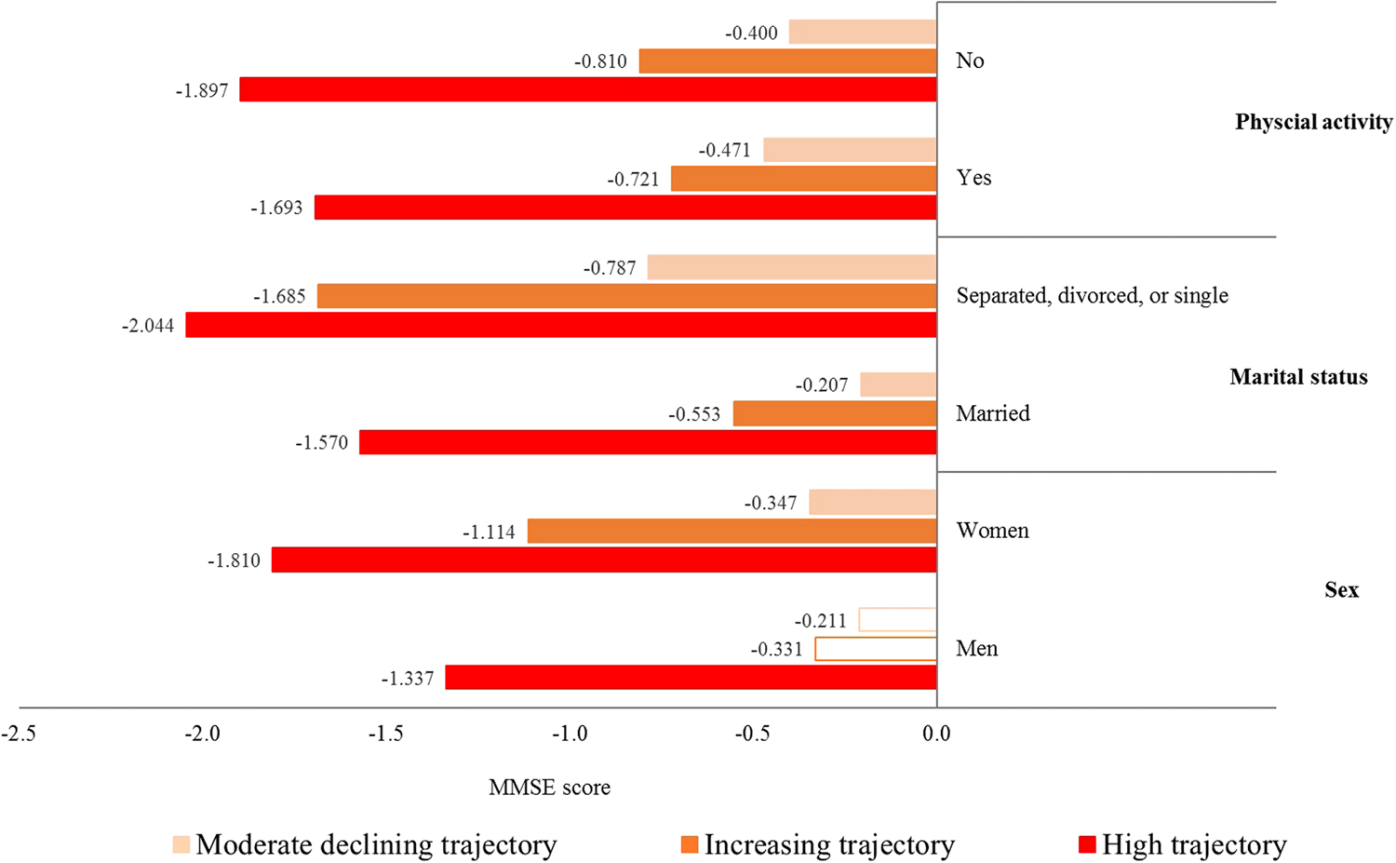
Results of the subgroup analysis for the association between the trajectory groups of depressive symptoms and MMSE scores in a mixed-effects model according to sex, marital status, and physical activity. ^†^Analysis was adjusted for the following covariates: sex, age, education level, household assets, employment status, marital status, BMI, physical activity, alcohol consumption, smoking, hypertension, and cerebrovascular disease. ^‡^The color bars indicate statistically significant results (*P* < 0.05).

Figure 2 presents the results of the subgroup analyses in which we assessed the relationship between the CES-D10 score trajectories and MMSE scores according to sex, marital status, and physical activity. We observed that women in the increasing (*β* = −1.114, *P* ≤ 0.001), moderate declining (*β* = −0.347, *P* = 0.011), and high (*β* = −1.810 *P* ≤ 0.001) CES-D trajectory groups had lower MMSE scores than did those in the low CES-D trajectory group. However, among men, only those in the high CES-D trajectory group had lower MMSE scores than those in the low CES-D trajectory group (*β* = −1.337, *P* ≤ 0.001). Among separated, divorced, or single participants, those with increasing (*β* = −1.685, *P* = 0.003), moderate declining (β = −0.787, *P* = 0.019), and high (*β* = −2.044, *P* ≤ 0.001) CES-D trajectories had lower MMSE scores than did those with a low CES-D trajectory. In participants who did not engage in physical activity, those with increasing (*β* = −0.810, *P* = 0.004), moderate declining (*β* = −0.400, *P* = 0.011), and high (*β* = −1.897, *P* ≤ 0.001) CES-D trajectories had lower MMSE scores than did those with a low CES-D trajectory (Table S1 in Supplementary).

**Figure 3:**
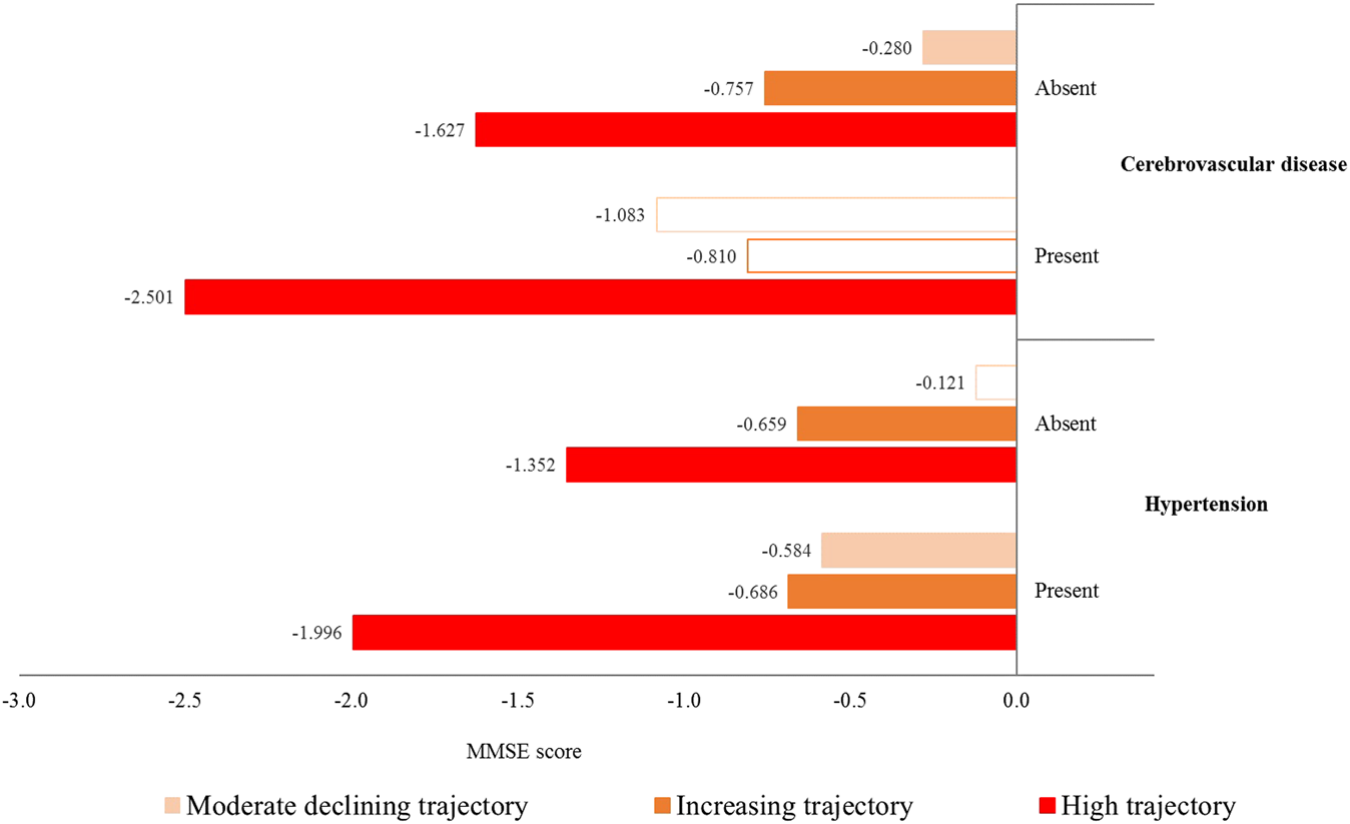
Results of the subgroup analysis for the association between the trajectory groups of depressive symptoms and MMSE scores in a mixed-effects model according to hypertension and cerebrovascular disease. ^†^Analysis was adjusted for the following covariates: sex, age, education level, household assets, employment status, marital status, BMI, physical activity, alcohol consumption, smoking, hypertension, and cerebrovascular disease. ^‡^The color bars indicate statistically significant results (*P* < 0.05).

Figure 3 presents the results of the subgroup analysis for the association between the trajectory groups of depressive symptoms and MMSE scores in a mixed-effects model according to hypertension and cerebrovascular disease. Among participants with hypertension, those with increasing (*β* = −0.810, *P* = 0.004), moderate declining (*β* = −0.400, *P* = 0.011), and high (*β* = −1.897, *P* ≤ 0.001) CES-D trajectories had lower MMSE scores than did those with a low CES-D trajectory. However, among the participants with cerebrovascular disease, only those in the high CES-D trajectory group had a lower MMSE score than those in the low CES-D trajectory group (*β* = −2.501, *P* = 0.027).

Figure S1 in Supplementary shows the association between the depressive-symptom trajectory groups and the MMSE scores in the follow-up groups of 2008–2010, 2010–2012, 2012–2014, and 2014–2016. Compared with the low trajectory group, the increasing and high trajectory groups had lower MMSE scores in 2008–2010 (increasing CES-D: *β* = −0.650, *P* ≤ 0.001; high CES-D: β = −1.222, *P* ≤ 0.001) and 2010–2012 (increasing CES-D: *β* = −0.923, *P* ≤ 0.001; high CES-D: *β* = −1.284, *P ≤ *0.001). During 2012–2014, the increasing (*β* = −0.891, *P* ≤ 0.001), moderate declining (*β* = −0.247, *P* = 0.042), and high (*β* = −1.585, *P* ≤ 0.001) trajectory groups had lower MMSE scores than did the low trajectory group. Between 2014–2016, only the moderate declining (*β* = −0.481, *P* ≤ 0.001) and high (*β* = −1.906, *P* ≤ 0.001) CES-D trajectory groups had lower MMSE scores than did the low trajectory group.

## Discussion

In this study, we identified four groups according to their trajectories of changes in depressive symptoms, which we classified as low, increasing, moderate declining, and high depressive-symptom trajectories. We observed that cognitive-function decrease was higher in the increasing, moderate declining, and high trajectory groups than that in the low trajectory group across the 8- year study period in this late middle-aged and older population. These relationships were particularly strong among women, physically-inactive individuals, separated, divorced, or single individuals; and those with hypertension or cerebrovascular disease, particularly if they had rapidly-increasing or chronically-high depressive symptoms. Moreover, these relationships were maintained across all follow-up periods (i.e., 2008–2010, 2010–2012, 2012–2014, and 2014–2016).

We suggest that each trajectory of depressive symptoms during the 8-year period could be useful to predict differential risk factors and to more accurately distinguish people at risk of cognitive decline. We observed that not only people in the high trajectory group but also those with a sharp increase in depressive symptoms had a high risk of cognitive-function decline. Moreover, participants in the increasing, moderate declining, and high depressive symptom trajectories tended to be older, of female sex, poorly educated, living alone, and physically inactive, as well as more likely to have hypertension or cerebrovascular disease compared with those in the low depressive-symptom trajectory. Previous studies have found that poor physical health status, involving, among others, chronic diseases and functional limitations, was related to an increase in the risk of depressive symptoms^20–22^. Similar characteristics of participants classified to each trajectory have also been reported by previous studies^16,20,23^. The MMSE scores in the increasing, moderate declining, and high depressive-symptom trajectory groups were lower by approximately 0.3 to 1.6 points than in the low depressive-symptom trajectory group. This further supports the assertion that the moderate declining, high and increasing trajectory groups were also at risk of decreased cognitive function.

Previous research has also suggested that participants with steadily-increasing depressive symptoms have a high risk of dementia^16^. Although the underlying mechanisms remain to be identified, it is known that elderly people with depression have more cerebral white matter and other subcortical abnormalities compared with healthy elderly people^24,25^. Neurodegenerative diseases, such as Alzheimer’s disease and vascular dementia, may begin in the frontal and limbic systems and may include the serotonin and noradrenaline pathways. Patients with these conditions may initially present with depressive symptoms^26,27^, such that depression may represent the early stages of a neurodegenerative disease. Therefore, people with high and rapidly-increasing depressive symptoms may likely be at risk of developing dementia.

To investigate the potential presence of reverse causality, we stratified the follow-up period into 2008–2010, 2010–2012, 2012–2014, and 2014–2016. In this analysis, we observed an identical trend of a significantly negative association between depressive symptoms and MMSE scores in all trajectory groups, except for the moderate declining trajectory group. In particular, there was a stronger relationship between depressive symptoms and cognitive-function decline in the participants in the high trajectory groups, who had chronic depressive symptoms, compared with those in the other trajectory groups, which was consistent across the investigated time periods. These results suggest that people with chronic depression are likely to be at higher risk of dementia and should be especially targeted for prevention and management.

In the subgroup analysis of sex, women had lower MMSE scores in the increasing, moderate declining, and high depressive-symptom trajectory groups compared with the low trajectory group. In contrast, among men, only those in the high depressive-symptom trajectory group had decreased MMSE scores compared with those in the low trajectory group. Moreover, the coefficients for all trajectories, but especially for the increasing trajectory, were larger in women than in men. This indicates that depressive symptoms had a greater effect on cognitive-function decline in women than in men.

It is possible that high levels of cortisol may be associated with depression and may also lead to neuronal death and cognitive decline. High cortisol levels or other manifestations of hypothalamic-pituitary-adrenal axis dysregulation are more common among women and might be associated with depressive symptoms and poor cognitive function. Such dysregulation may account for the more severe cognitive decreases seen in women. It is also possible that depressive symptoms and cognitive decline are both attributable to an underlying genetic predisposition^27–29^.

We also observed that people who were separated, divorced, or single had greater MMSE score declines than did those who were married in all depressive-symptom trajectories, but especially the increasing and high trajectory groups. This is consistent with the findings of a previous study in which it was observed that married people had a low risk of MMSE decline^30^. Strong social networks may protect against cognitive decline. Therefore, being married may represent being part of a family social network, which can help reduce declining cognitive function^31^.

Moreover, the physical-inactive group showed a strong association with decline in cognitive function in all trajectories of depressive symptoms compared to the physical-active group. Physical activity has been reported to both postpone cognitive-function decline and to prevent depression^32,33^. Although the mechanisms of these benefits are not clear, some possibilities include an alteration in cerebral vascular functioning and brain perfusion or environment enrichment associated with greater physical activity^34,35^. Thus, we suggest that people who are physically inactive should be encouraged to increase the time they spend engaging in physical activities, as this would benefit their mental health, as well as help avoid neurodegenerative disease.

Both hypertension and cerebrovascular disease have been related to worsened cognitive performance^36,37^. In our study, participants with hypertension or cerebrovascular disease in the high depressive-symptom trajectory group had greater declines in cognitive function than did those without these conditions. Although participants with hypertension in all trajectory groups showed a strong relationship between depressive symptoms and cognitive decline, for participants with cerebrovascular disease, this strong association was only seen among those in the high trajectory group. Nonetheless, these results suggest that patients with these conditions should be supported to effectively manage their physical and mental health to postpone the onset of cognitive decline.

This study is subject to several limitations. First, despite the use of the CES-D10 and MMSE, the participant scores may have been subjective or erroneous. Second, although our trajectory model was fitted based on assigned trajectories, we did not fully consider the uncertainty of class membership of each participant, which signifies that the variance estimates from this model are likely to be underestimated. Third, health behaviors such as alcohol consumption, smoking, and physical activity were not assessed with specific scales. Fourth, we could not fully assess sex differences for patterns of depressive-symptom trajectories and cognitive function. Further studies are needed to evaluate pertinent sex differences. Fifth, we cannot exclude the possibility that the associations found in our analysis may in fact be due to cohort effects due to the unstructured, multicohort, longitudinal design of the study and the broad age range of the participants^38^. This study included participants 60 years or older at baseline, who experienced the Korean War in 1950–1953, as well as “baby boomer” participants in their 40’s and 50’s at baseline, whose life experiences were markedly different. The different life experiences of these cohorts may have had unknown effects on depressive symptoms and cognitive function^39^. Therefore, the results should be interpreted carefully, and further research needs to consider this effect. Finally, we may not have fully controlled for all potential risk or confounding factors in this study. However, despite these limitations, our study has several strengths. First, the study had a relatively long follow up and used a representative elderly sample. Second, we investigated both the long- and short-term effects of depressive symptoms on cognitive-function decline using different courses of depressive symptoms with respect to increased risk of dementia. Finally, we stratified the follow-up period from 2008 to 2016 for each 2 years to explore the possibility of reverse causal relations.

In conclusion, participants in all depressive-symptom trajectory groups, classified by repeated measurements, showed decreased MMSE scores over the follow-up period. However, women, the physically inactive, and those with hypertension or cerebrovascular disease had worse cognitive function, suggesting a need to manage the mental health of such individuals through psychiatric consultation or by visiting clinics for the prevention of dementia, particularly if they have rapidly-increasing or chronically-high depressive symptoms. Public health professionals and policymakers should consider our findings when developing optimal health policies for the prevention of dementia.

## Supplementary information

Association between depressive symptoms trajectories and cognitive function in late middle-aged and older population: results of the Korean Longitudinal Study of Ageing
