# Supplementary material for: Association between depressive-symptom trajectories and cognitive function in the late middle-aged and older population: results of the Korean Longitudinal Study of Ageing
Source: Sci Rep. 2019 May 24;9:7807. doi: 10.1038/s41598-019-44158-7 (PMC6534670; doi:10.1038/s41598-019-44158-7)
Supplement: Supplementary file 1 — Association between depressive symptoms trajectories and cognitive function in late middle-aged and older population: results of the Korean Longitudinal Study of Ageing [file 41598_2019_44158_MOESM1_ESM.docx]

**Association between depressive-symptom trajectories and cognitive function in the late middle-aged and older population: results of the Korean Longitudinal Study of Ageing**

Dong-Woo Choi, BA^*1,2^: cdw6027@yuhs.ac

Kyu-Tae Han, PhD^*3^: kthan.phd@gmail.com

Jooeun Jeon, MPH^1^: jejeon@yuhs.ac

Sung-In Jang, MD, PhD^2,4^: Jangsi@yuhs.ac

Seung Ju Kim, PhD^5^: seungju.phd@gmail.com

Eun-Cheol Park, MD, PhD^**,2,4^: ecpark@yuhs.ac

^1^ Department of Public Health, Graduate School, Yonsei University, Seoul, Republic of Korea

^2^ Institute of Health Services Research, Yonsei University, Seoul, Republic of Korea

^3^ Hospice & Palliative Care Branch, National Cancer Control Institute, National Cancer Center, Goyang, South Korea

^4^ Department of Preventive Medicine, Yonsei University College of Medicine, Seoul, Republic of Korea

^5^ Department of Nursing, College of Nursing, Eulji University, Seongnam, Gyeonggi-do, Republic of Korea

*** These authors contributed equally to this work.**

**** Corresponding author**: Eun-Cheol Park, MD, PhD

Department of Preventive Medicine & Institute of Health Services Research, Yonsei University College of Medicine

50-1 Yonsei-ro, Seodaemun-gu, Seoul 03722, Republic of Korea

Tel: 82-2-2228-1862 Fax: 82-2-392-8133 E-mail: ecpark@yuhs.ac

**
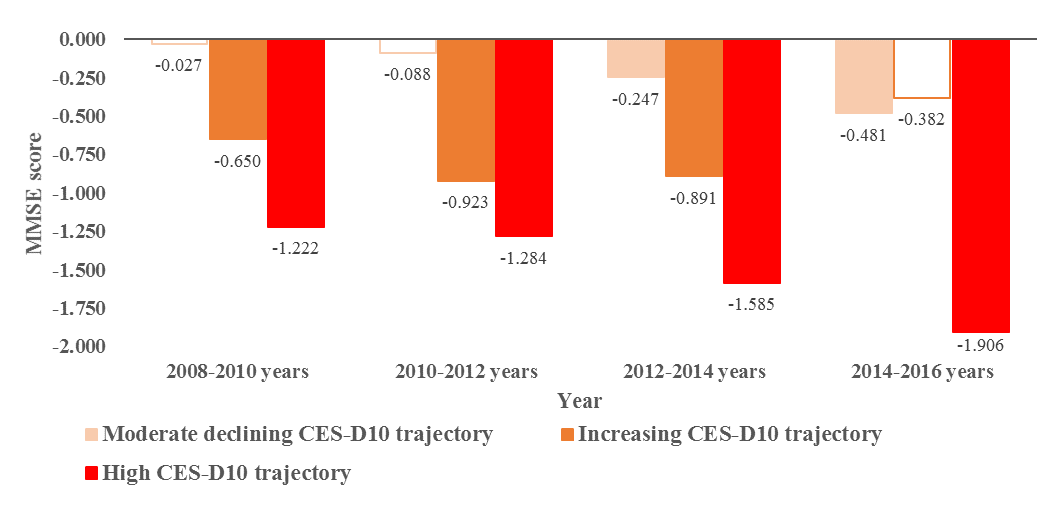
**

**Figure S1. The association between depressive symptom trajectory groups and MMSE scores in the follow-up groups of 2008–2010, 2010–2012, 2012–2014, and 2014–2016.**

† Analysis was adjusted for the following covariates: sex, age, education level, household assets, employment status, marital status, BMI, physical activity, alcohol consumption, smoking, hypertension, and cerebrovascular disease.

‡ The color bars indicate statistically significant results (P < 0.05).

**Table S1. Results associated with MMSE scores in a mixed-effects regression analysis**

| **Variables** | **MMSE score** | | |
| --- | --- | --- | --- |
|  | **β** | **SE** | **P-value** |
| **CES-D10 score** | -0.124 | 0.009 | <.001 |
| **Trajectory groups** |  |  |  |
| Low CES-D10 trajectory | Ref. |  |  |
| Moderate declining CES-D10 trajectory | -0.278 | 0.094 | 0.003 |
| Increasing CES-D10 trajectory | -0.729 | 0.173 | <.001 |
| High CES-D10 trajectory | -1.605 | 0.119 | <.001 |
| **Sex** |  |  |  |
| Men | 0.179 | 0.103 | 0.082 |
| Women | Ref. |  |  |
| **Age (years)** |  |  |  |
| 45 - 54 | Ref. |  |  |
| 55 - 64 | 0.071 | 0.070 | 0.306 |
| 65 - 74 | -0.442 | 0.092 | <.001 |
| ≥75 | -1.616 | 0.119 | <.001 |
| **Educational level** |  |  |  |
| Elementary school or under | -2.072 | 0.134 | <.001 |
| Middle school | -0.758 | 0.138 | <.001 |
| High school | -0.407 | 0.126 | 0.001 |
| University or above | Ref. |  |  |
| **Household assets quartile** |  |  |  |
| 1st quartile | -0.292 | 0.086 | <.001 |
| 2nd quartile | -0.352 | 0.074 | <.001 |
| 3rd quartile | -0.097 | 0.062 | 0.117 |
| 4th quartile | Ref. |  |  |
| **Employment status** |  |  |  |
| Employed | Ref. |  |  |
| Unemployed | -0.311 | 0.058 | <.001 |
| **Marital status** |  |  |  |
| Married | Ref. |  |  |
| Separated, divorced, or single | -0.278 | 0.085 | 0.001 |
| **BMI** |  |  |  |
| Pre-obesity or obesity | 0.209 | 0.069 | 0.003 |
| Overweight | 0.251 | 0.056 | <.001 |
| Normal or underweight | Ref. |  |  |
| **Physical activity** |  |  |  |
| Yes | 0.513 | 0.047 | <.001 |
| No | Ref. |  |  |
| **Alcohol behavior** |  |  |  |
| Drinker | 0.293 | 0.082 | <.001 |
| Ex-drinker | -0.234 | 0.101 | 0.020 |
| Non-drinker | Ref. |  |  |
| **Smoking behavior** |  |  |  |
| Current smoker | -0.079 | 0.106 | 0.459 |
| Ex-smoker | 0.117 | 0.109 | 0.283 |
| Non-smoker | Ref. |  |  |
| **Hypertension** |  |  |  |
| Present | -0.267 | 0.064 | <.001 |
| Absent | Ref. |  |  |
| **Cerebrovascular disease** |  |  |  |
| Present | -1.339 | 0.149 | <.001 |
| Absent | Ref. |  |  |
| **Year** | 0.047 | 0.015 | 0.003 |

**Table S2. Means and standard deviation of MMSE score according to CESD trajectories and years**

| **Variables** | **Low CES-D10  trajectory** | | | **Moderate declining CES-D10  trajectory** | | | | **Increasing CES-D10  trajectory** | | | | **High CES-D10  trajectory** | | | |
| --- | --- | --- | --- | --- | --- | --- | --- | --- | --- | --- | --- | --- | --- | --- | --- |
|  | **Means** | **SD** | **P-value** | | **Means** | **SD** | **P-value** | | **Means** | **SD** | **P-value** | | **Means** | **SD** | **P-value** |
| **Years** |  |  | <.001 | |  |  | <.001 | |  |  | <.001 | |  |  | <.001 |
| 2008 (wave 2) | 27.7 | 2.6 |  | | 27.0 | 3.0 |  | | 27.2 | 2.7 |  | | 25.6 | 3.2 |  |
| 2010 (wave 3) | 26.0 | 2.5 |  | | 25.2 | 2.9 |  | | 25.4 | 3.1 |  | | 23.7 | 3.7 |  |
| 2012 (wave 4) | 27.0 | 2.8 |  | | 25.6 | 4.0 |  | | 26.3 | 3.2 |  | | 24.5 | 4.2 |  |
| 2014 (wave 5) | 27.4 | 3.2 |  | | 26.3 | 4.2 |  | | 26.6 | 4.0 |  | | 24.3 | 4.7 |  |
| 2016 (wave 6) | 27.7 | 3.1 |  | | 26.4 | 3.9 |  | | 26.3 | 4.2 |  | | 23.8 | 5.2 |  |
